# MicroRNA-26a promotes anoikis in human hepatocellular carcinoma cells by targeting alpha5 integrin

**DOI:** 10.18632/oncotarget.2956

**Published:** 2014-12-10

**Authors:** Xiang Zhang, Shu-Li Cheng, Ka Bian, Lei Wang, Xiao Zhang, Bo Yan, Lin-Tao Jia, Jing Zhao, Noor Gammoh, An-Gang Yang, Rui Zhang

**Affiliations:** ^1^ State Key Laboratory of Cancer Biology, Department of Biochemistry and Molecular Biology, the Fourth Military Medical University, Xi’an, Shaanxi, China; ^2^ Department of Orthodontics, School of Stomatology, the Fourth Military Medical University, Xi’an Shaanxi, China; ^3^ Department of Otolaryngology, Tangdu Hospital, the Fourth Military Medical University, Xi’an Shaanxi, China; ^4^ State Key Laboratory of Cancer Biology, Department of Immunology, the Fourth Military Medical University, Xi’an, Shaanxi, China; ^5^ Edinburgh Cancer Research Centre, The University of Edinburgh, Western General Hospital, Edinburgh EH4 2XR, United Kingdom

**Keywords:** microRNA-26a, ITGA5, human hepatocellular carcinoma, anoikis, tumor metastasis

## Abstract

Metastasis is the major reason for the death of patients suffering from malignant diseases such as human hepatocellular carcinoma (HCC). Among the complex metastatic process, resistance to anoikis is one of the most important steps. Previous studies demonstrate that microRNA-26a (miR-26a) is an important tumor suppressor that inhibits the proliferation and invasion of HCC cells by targeting multiple oncogenic proteins. However, whether miR-26a can also influence anoikis has not been well established. Here, we discovered that miR-26a promotes anoikis of HCC cells both *in vitro* and *in vivo.* With a combinational analysis of bioinformatics and public clinical databases, we predicted that alpha5 integrin (ITGA5), an integrin family member, is a putative target of miR-26a. Furthermore, we provide experimental evidence to confirm that ITGA5 is a *bona fide* target of miR-26a. Through gain- and loss-of-function studies, we demonstrate that ITGA5 is a functional target of miR-26a-induced anoikis in HCC cells. Collectively, our findings reveal that miR-26a is a novel player during anoikis and a potential therapeutic target for the treatment of metastatic HCC.

## INTRODUCTION

Hepatocellular carcinoma (HCC) is among the most prevalent and lethal cancers with continuously rising incidence and a 5-year survival rate of less than 12% [1, 2]. Despite of the great advances in current therapeutics, relapse and metastasis are still frequently observed in the clinic highlighting the need to develop novel therapeutic strategies for HCC [3]. Therefore, in depth understanding of the complex network of HCC is a prerequisite to develop effective treatment.

Unlike well-defined primary tumors, metastatic cancers are incurable due to their systemic and surgically inoperable characteristics and account for more than 90% of cancer-associated mortality [4]. Metastases arise through the completion of a series of successive cell-biological events termed the invasion-metastasis cascade, in which dissemination of tumor cells to anatomical distant organ sites is one of the most pivotal steps [5]. Anoikis is a kind of detachment-induced apoptosis, and anoikis-resistance allows survival of tumor cells during systemic circulation, consequently facilitating secondary tumor formation in distant organs [6]. The aberrant high activity of multiple signaling pathways, including integrin signaling pathway has been demonstrated to be required to prevent anoikis in tumor cells [7, 8]. And blockage of anti-anoikis activity of tumor cells has been shown to be beneficial for the treatment of metastatic diseases [6, 7]. Each integrin consists of one α and β subunits. In mammalian cells, 18 α and 8 β subunits associate in various combinations to form 24 integrins. They transmit signals to exert a stringent control on cell survival, proliferation, adhesion and migration [9]. Especially recently, it has been demonstrated that the aberrantly high activation of integrins plays a critical role for metastasis of human hepatocellular carcinoma cells [10].

MicroRNAs (miRNAs), a class of endogenous 21- to 25- nucleotide highly conserved non-coding RNAs, play a critical role in a variety of normal developmental and physiological processes [11]. They negatively regulate gene expression in a posttranscriptional manner through directly degrading messenger RNA (mRNA) or indirectly repressing translation [12]. A growing body of evidence demonstrates that dysregulation of miRNAs frequently occurs during tumor initiation and progression [13]. Recent results document functional contributions of speciﬁc miRNAs to cellular transformation and tumorigenesis. According to their biological functions, tumor-associated miRNAs are classified as tumor suppressor miRNAs and oncomiRs [14]. In HCC, a series of tumor suppressor miRNAs have been confirmed as a potent “magic bullet” in cancer gene therapy [15]. For example, downregulation of miR-124 is reported in an inflammation-induced feedback circuit of HCC cells [16]. Administration of miR-124 mimics attenuates inflammatory signaling, prevents and suppresses hepatocellular carcinogenesis by inducing tumor-specific apoptosis [16]. And recently, Zhuang's group reported that miR-100 functions as anti-metastatic microRNA in HCC cells by blocking the ICMT-Rac1 signaling [17]. Furthermore, our recent study showed that miR-101, a c-Myc and PRC2 epigenetic repressor complex synergetically silenced miRNA, exerts as a potential tumor suppressor in HCC by targeting multiple oncogenic pathways [18]. Strikingly, miR-26a, another well-documented tumor suppressor miRNA in HCC, is also demonstrated to be involved in a c-Myc and EZH2-controlled molecular network in lymphoma, prostate cancer and colorectal cancer [19-22]. All evidence supports that tumor suppressor miRNAs could be a key target in developing effective treatment against HCC. Data from several large cohort studies showed that miR-26a is frequently silenced in HCC tissues and can act as a prognosis factor [23]. Moreover, adeno-associated virus (AAV)-mediated systematic administration of miR-26a potentially inhibits tumor growth in a spontaneous murine liver cancer model [24]. However, it is unclear how the currently identified target genes of miR-26a contribute to its strong inhibitory effects on the malignant behaviors of HCC cells. Additional studies are still required to identify the mechanism of tumor suppression by miR-26a thereby shedding light on the complex molecular network of HCCs.

In this study, we discovered that overexpression of miR-26a promotes anoikis of HCC cells *in vitro* and *in vivo*. We predicted putative target genes of miR-26a by bioinformatics, and further analysed these genes in public clinical databases. We identified ITGA5, an integrin family member, as a *bona fide* functional target in miR-26a-induced anoikis. Collectively, our study provides a possible regulatory pathway for ITGA5 and a candidate promoter of anoikis of HCC cells.

## RESULTS

### The miR-26a expression is significantly downregulated in HCC cells and especially in metastatic tumors

**Figure 1:**
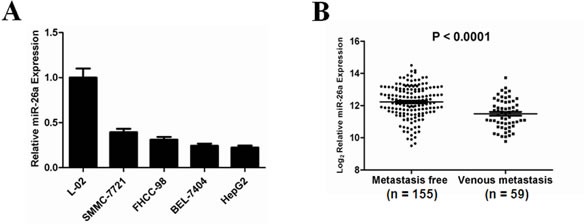
Down-regulation of miR-26a expression in human HCC, especially in metastatic HCC tumors (A) Relative expression levels of miR-26a (mean ± s.d. of three independent experiments) assessed by qRT-PCR in normal hepatocyte-derived cell line (L-02) and various HCC cell lines. (B) Comparison of the expression levels of miR-26a between metastasis-free and metastatic HCC. Expression levels of total miR-26a as the average expression levels of pre-miR-26a-1 and pre-miR-26a-2 from a normalized GEO dataset (GSE6857). Vertical axes represent log (base 2) relative quantification values. *P* value indicates statistical significance analyzed by Student's *t* test.

To investigate the role of miR-26a in human HCC, we detected the expression levels of miR-26a in a panel of human HCC cell lines and a hepatocyte-derived immortal cell line by using qRT-PCR. The results in Figure 1A show remarkably lower levels of miR-26a in HCC cells compared to an immortalized liver cell line, in agreement with a tumor suppressor role of miR-26a in HCC. To further confirm whether miR-26a is associated with metastasis of HCC, a normalized Gene Expression Omnibus (GEO) dataset (GSE6857) was analyzed. The data show significant downregulation of miR-26a in venous metastatic cancer tissues compared to metastasis-free cancer tissues (Figure 1B). These observations are in agreement with previous findings that miR-26a expression is down-regulated in parallel with hepatocellular cancerization and metastasis [23-26].

### Overexpression of miR-26a promotes HCC cells anoikis *in vitro* and *in vivo*

**Figure 2:**
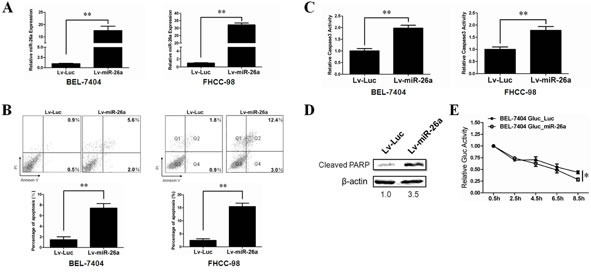
Stable over-expression of miR-26a promotes cell anoikis *in vitro* and *in vivo* (A) Relative miR-26a expression levels in BEL-7404(left) and FHCC-98(right) cells treated with Lv-Luc or Lv-miR-26a were determined by qRT-PCR. (B, C) The anoikis activity of BEL-7404(left) and FHCC-98(right) cells treated with Lv-Luc or Lv-miR-26a was evaluated by Annexin-V/PI staining (B) and caspase-3 activity(C). Cells (1×10^6^) were cultured for 48 h in poly-HEMA pre-coated plates before evaluation. (D) Western blot analysis was performed to determine the cleavage of PARP in FHCC-98 cells treated with Lv-Luc or Lv-miR-26a. Cells (1×10^6^) were cultured for 48 h in poly-HEMA pre-coated plates before evaluation. (E) BEL-7404 Gluc stably miR-26a expressed cells and control cells (1×10^6^) were injected into nude mice (n=5) through tail vein. Gluc activity analysis was performed to determine the anoikis activity of different cell lines *in vivo*. **, *P* < 0.01. *, *P* < 0.05. *Error bars*, s.d. (experiments depicted in A-C performed in triplicate).

Metastasis involves a series of sequential stages and the inhibition of anoikis plays a critical role in this complicated process [7]. The biological effect and mechanism of miR-26a on tumor cell anoikis has not been clarified. To determine a role of miR-26a in anoikis, we used lentivirus-delivery system to stably overexpress miR-26a in two HCC cell lines (BEL-7404 and FHCC-98) and confirmed the expression level of miR-26a by qRT-PCR analysis (Figure 2A). Tumor cells were seeded on plates pre-coated with polyHEMA that prevent cell attachment. After 48h, flow cytometry was performed to measure the percentage of dying cells. As shown in Figure 2B, overexpression of miR-26a clearly induced more cell death as compared with a control group. The induction of caspase-3 activity and PARP cleavage further confirmed that miR-26a-induced cell death occurs mainly through cell-detachment induced apoptosis (Figure 2C, 2D).

Based on our discovery *in vitro*, we then asked whether miR-26a can affect anoikis of HCC cells *in vivo*. Considering that anoikis is a transient process in circulating tumor cells and that there is only a narrow time frame to detect it *in vivo* [27], we expressed *Gaussia* Luciferase in the HCC cell line BEL-7404. Gaussia luciferase (Gluc) is a naturally secreted luciferase from the marine copepod Gaussia princeps. It is over thousands of folds more sensitive than firefly and renilla luciferases in mammalian cells [28]. Since Gluc is secreted, its concentration in the blood correlates with expression level in an *in vivo* system. Theoretically, the blood Gluc assay can reveal survival and early growth of metastatic tumors before BLI could visualize their presence [29]. This allows for convenient quantitative measurement of HCC cells sensitivity to anoikis *in vivo.* Using tail vein injection, tumor cells were administrated into nude mice. Blood samples were collected at the indicated time points and the Gluc activity was measured. The data showed that overexpression of miR-26a significantly decreased the Gluc activity in the circulating system of nude mice, indicating that miR-26a potentially sensitizes anoikis of tumor cell *in vivo* (Figure 2E).

### ITGA5 is a bona fide target gene of miR-26a

**Figure 3:**
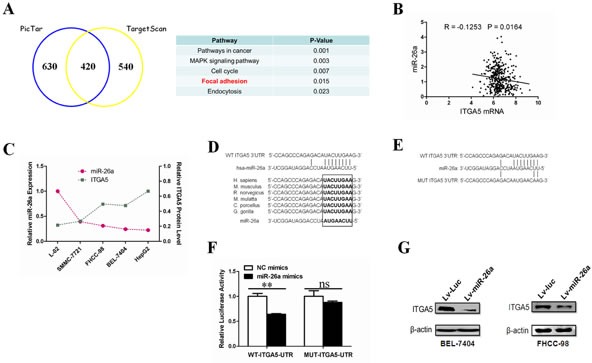
ITGA5 is a bona fide target gene of mir-26a (A) Gene Ontology classification of miR-26a's potential target genes predicted by integrating the results of two algorithms (Targetscan and PicTar). (B) The correlation of ITGA5 mRNA and miR-26a in human hepatocellular cancer tissues. The Pearson product-moment correlation coefficient and significance levels are indicated. Expression levels of ITGA5 and miR-26a were from two correlative normalized GEO datasets (GSE14520 & GSE6857). (C) The correlation between miR-26a expression and the levels of ITGA5 in different human HCC cell lines and normal hepatocyte-derived cell line (L-02). The miRNA expression was evaluated by qRT-PCR and protein abundance was evaluated by western blot. (D) Schematic diagram of the miR-26a target site of human and other representative mammal ITGA5 3′UTRs. (E) The wild-type 3′UTR of ITGA5 and mutant 3′UTR sequences that abolished binding. (F) A reporter vector containing the WT(wild-type) or MUT(mutant) ITGA5 3′UTR was transfected, along with miR-NC or miR-26a, into HEK-293 cells. Luciferase activity was measured in three independent experiments after 48 hours of transfection and normalized to Renilla luciferase activity. (G) Western blot analysis was performed to determine ITGA5 protein expression post infection with Lv-Luc or Lv-miR-26a. **, *P* < 0.01. NC, negative control. ns, no significance. Luc, luciferase.

To identify the effectors of miR-26a-induced anoikis, we used combined analyses of TargetScan (http://www.targetscan.org/) and PicTar (http://pictar.mdc-berlin.de/) databases to predict the putative target genes of miR-26a (Figure 3A). Using DAVID Bioinformatics Resources (http://david.abcc.ncifcrf.gov/), gene ontology (GO) analysis revealed that the candidate genes were functionally enriched in several biological processes (Figure 3A). We focused on genes related to the focal adhesion pathway because of its close relationship with anoikis as reported previously [30]. The putative target genes in the focal adhesion pathway were COL1A2, COL5A1, PDGFRA, ITGA5 and ITGA6 (Figure S1). Since a previous study has demonstrated that the most target genes of miRNAs are regulated in the mRNA level [31], to further narrow down the candidates, we performed *in silico* studies. Analyses of two normalized GEO datasets (GSE14520 & GSE6857) show that only ITGA5 mRNA levels were inversely correlated with miR-26a (Figure 3B, Figure S2). Furthermore, we examined the expression levels of miR-26a and ITGA5 in a panel of human HCC cell lines. qRT-PCR and Western blotting analyses revealed a significant (*P* < 0.05) inverse correlation between miR-26a and ITGA5 expression levels (Figure 3C, Figure 1A and Figure S3). The seed sequence of miR-26a is complementary to the 3′UTR of ITGA5 and is highly conserved in six different species (Figure 3D). These findings indicate that ITGA5 is a potential target of miR-26a.

Next, we investigated whether the 3′UTR of ITGA5 is a direct target of miR-26a. To do so, we cloned the 3′UTR of ITGA5 harboring the complementary sequence to miR-26a seed sequence in a reporter plasmid vector. In parallel, we mutated the 3′UTR of ITGA5 complementary to the miR-26a seed sequence in the same reporter plasmid (Figure 3E). Transient transfection of HEK-293 cells with the ITGA5-3′UTR construct along with miR-26a led to a significant decrease in reporter expression when compared with the control samples (Figure 3F). The luciferase activity of the reporter vector containing a mutated 3′UTR of ITGA5 was unaffected by simultaneous transfection of miR-26a (Figure 3F). These results suggest that miR-26a directly binds and negatively regulates ITGA5 mRNA stability. We then sought to determine whether exogenous miR-26a can regulate ITGA5 expression in HCC cells. As shown in Figure 3G, overexpression of miR-26a strongly inhibits ITGA5 protein levels. Taken together, these results support the notion that ITGA5 is a *bona fide* target gene of miR-26a in HCC cells.

### Silencing ITGA5 recapitulates the effects of miR-26a on anoikis in HCC cells *in vitro* and *in vivo*

**Figure 4:**
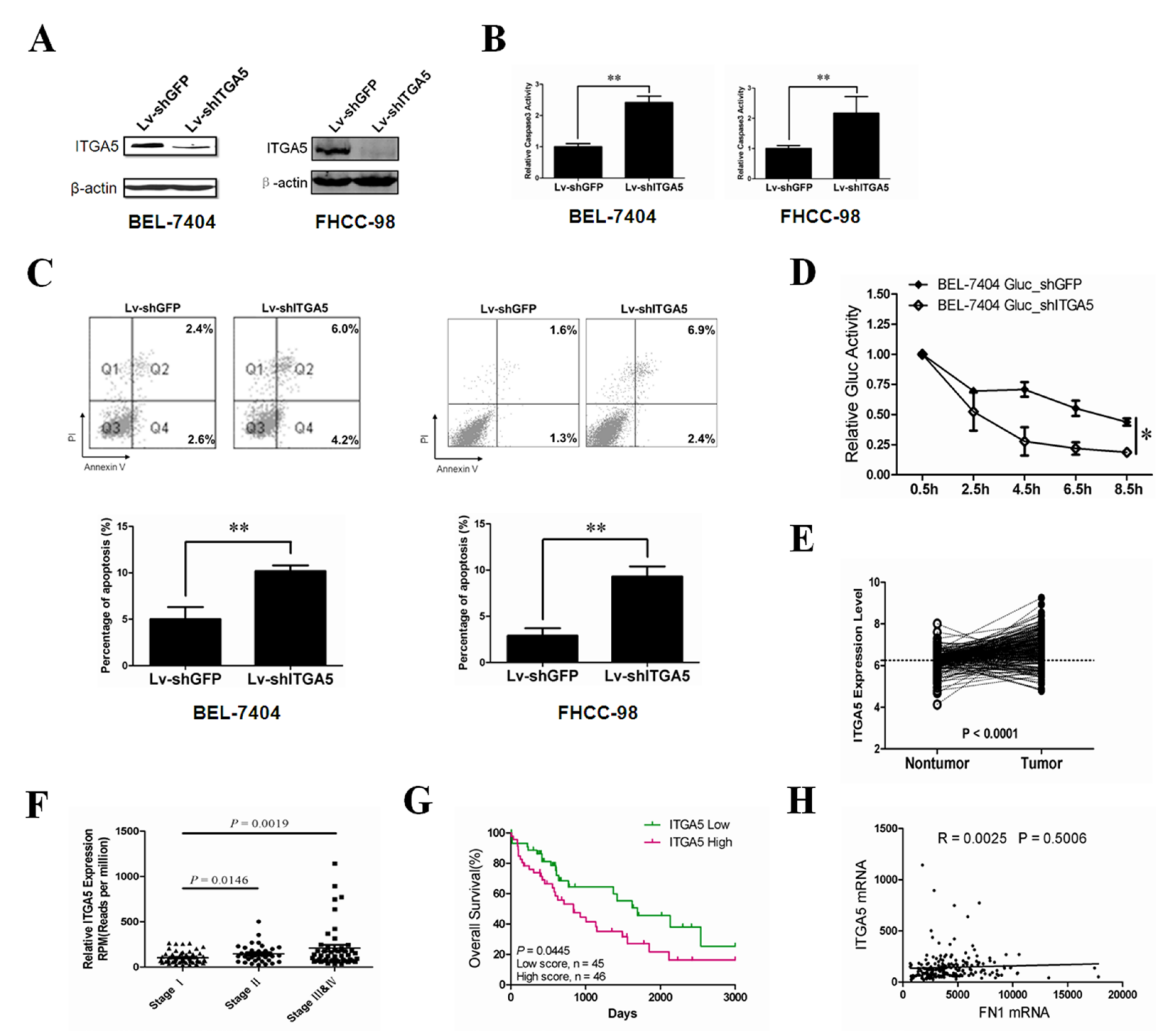
Functions of ITGA5 in HCC (A) Western blot analysis was performed to determine ITGA5 protein expression post-infection with Lv-shGFP or Lv-shITGA5. (B, C) The anoikis activity of BEL-7404(left) and FHCC-98(right) cells treated with Lv-shGFP or Lv-shITGA5 was evaluated by caspase-3 activity(B) and Annexin-V/PI staining(C). Cells (1×10^6^) were cultured for 48 h in poly-HEMA pre-coated plates before evaluation. (D) BEL-7404 Gluc stably ITGA5 knockdown cells and control cells (1×10^6^) were injected into nude mice (n=5) through tail vein. Gluc activity analysis was performed to determine anoikis in different HCC cell lines *in vivo*. (E) Comparisons of ITGA5 expression levels in paired tumor and non-tumor samples from 213 HCC patients, with the use of Student's *t* test; dashed line is shown at the mean of non-tumor group. Expression levels of ITGA5 were from a normalized GEO dataset (GSE14520). (F) Relative ITGA5 levels assessed by Deep Sequencing in HCC tissues stratified according to the stages of HCC. (G) Kaplan-Meier graph representing the probabilities of overall survival in HCC patients stratified according to the expression levels of ITGA5. (H) The correlation of ITGA5 and fibronection 1 (FN1) mRNA in human HCC tissues. The Pearson product-moment correlation coefficient and significance levels are indicated. In, expression levels of ITGA5 and FN1 were downloaded from the TCGA Liver hepatocellular carcinoma (LIHC) mRNA dataset. **, *P* < 0.01. *, *P* < 0.05. *Error bars*, s.d. (experiments depicted in B, C performed in triplicate).

ITGA5 is a member of the integrin family mediating cell-to-cell adhesion and can drive migration in tumor cells [32]. To investigate the role of ITGA5 on anoikis in HCC cells, we stably silenced endogenous ITGA5 using a lentivirus delivery system. ITGA5 expression was reduced by more than 90% (Figure 4A). Analysis of the cell detachment assay shows that knockdown of ITGA5 significantly increased anoikis of HCC cells *in vitro* (Figure 4B-C). Enhanced anoikis sensitivity by ITGA5 silencing mimicked the phenotype induced by overexpression of miR-26a in HCC cells. Furthermore, in our *in vivo* assay, silencing endogenous ITGA5 dramatically promoted tumor cell anoikis upon tail vein injection in nude mice (Figure 4D). Our results suggest that reduction of ITGA5 levels has similar effects on the HCC cells to miR-26a overexpression, further confirming that ITGA5 may act as a downstream functional mediator of miR-26a during tumor cell anoikis.

In order to further confirm the role of ITGA5 in HCC, we analysed its expression in clinical samples. A normalized GEO dataset mentioned above (GSE14520) indicated significantly higher levels of ITGA5 in HCC tissues as compared to normal tissues (Figure 4E). Moreover, the expression of ITGA5 significantly increased during the progression of HCC as indicated in the data derived from The Cancer Genome Atlas (TCGA) databases (Figure 4F). Our analysis of the HCC samples in TCGA databases also revealed that a high level of ITGA5 expression correlate with poor prognosis of patients (Figure 4G). The similar phenomenon was also detected in colorectal cancers (Figure S4). These analyses provide significant evidence that ITGA5 can drive oncogenesis in solid tumors.

It is well documented that ITGA5 and integrin β1 (ITGB1) form heterodimers to mediate cell adhesion to fibronectin (FN). Unexpectedly, we discovered that there is no obvious correlation between ITGA5 and FN expression level in human hepatocellular carcinomas (Figure 4H). This suggests that ITGA5 may have some uncharacterized functions to maintain survival of tumor cells, which is not associated with FN.

### Reconstituted activation of ITGA5 inhibits miR-26a-induced anoikis in HCC cells

**Figure 5:**
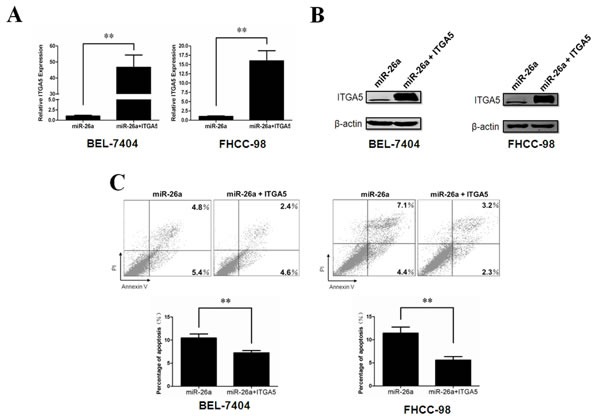
ITGA5 over-expression rescues phenotypes induced by miR-26a (A, B) Relative ITGA5 expression levels in BEL-7404(left) and FHCC-98(right) cells stably expressing ITGA5 were determined by qRT-PCR(A) and western blot(B). (C) The anoikis activity of ITGA5 stably over-expressed HCC cell lines was evaluated by Annexin-V/PI staining. Cells (1×10^6^) were cultured for 48 h in poly-HEMA pre-coated plates before evaluation. **, *P* < 0.01. *Error bars*, s.d. (experiments depicted in A&C performed in triplicate).

Our data suggest that ITGA5 is targeted by miR-26a during anoikis. Therefore, we predict that reconstitution of ITGA5 in miR-26a–expressing cells can antagonize the effects of miR-26a in this process. To test this, we infected miR-26a-expressing cells with lentivirus-delivered ITGA5 lacking the 3′UTR (Figure 5A-B). As expected, Annexin-V staining shows that reintroduction of ITGA5 counteract anoikis induced by miR-26a (Figure 5C). Taken together, our findings show that ITGA5 reintroduction can abrogate miR-26a–induced anoikis, suggesting that ITGA5 is a functional target of miR-26a in HCC cells.

### Inactivation of Akt is an essential downstream signal in ITGA5 suppression-induced tumor cell anoikis

**Figure 6:**
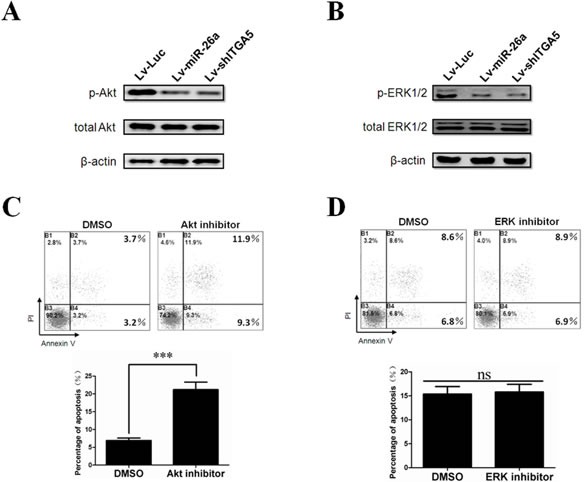
Inactivation of Akt is an essential downstream signal in ITGA5 suppression induced tumor cell anoikis (A) Western blot analysis was performed to determine the phosphorylation of Akt(Ser473) in BEL-7404 cells after over-expression of miR-26a or knockdown of ITGA5. (B) Western blot analysis was performed to determine the phosphorylation of ERK1/2(Thr202/Tyr204) in BEL-7404 cells after over-expression of miR-26a or knockdown of ITGA5. (C) The anoikis activity of BEL-7404 cells treated with DMSO or Akt inhibitor (15 μM) was evaluated by Annexin-V/PI staining. (D) The anoikis activity of BEL-7404 cells treated with DMSO or ERK inhibitor (5 μM) was evaluated by Annexin-V/PI staining. In &D, cells (1×10^6^) were cultured for 48 h in poly-HEMA pre-coated plates before evaluation. ***, *P* < 0.001. *Error bars*, s.d. ns, no significance. Luc, luciferase.

Our study suggests that miR-26a induces anoikis of HCC cells negatively regulating ITGA5. However, the downstream molecular events of how anoikis is regulated are still elusive. Previous data show that Akt and mitogen-activated protein kinase (MAPK, also known as EKR1/2) signaling networks play important roles in the malignant behavior of tumor cells [33, 34]. Therefore, we tested whether miR-26a-ITGA5 effects on anoikis acts through the Akt and ERK1/2 pathways. As shown in Figure 6A-B, the upregulation of miR-26a and downregulation of ITGA5 resulted in reduced phosphorylation of Akt and ERK1/2, whereas the total Akt and ERK levels were not affected, indicating both Akt and ERK1/2 signaling pathways could be the downstream of miR-26-ITGA5 axis. Intriguingly, pharmacological inhibition of the Akt pathway, but not the ERK pathway, resulted in a significant increase of anoikis in tumor cells (Figure 6C-D). This suggests that the ERK1/2 pathway is dispensable during anoikis of HCC cells in our system. Collectively, these ﬁndings demonstrated that Akt inactivation is an essential downstream signaling pathway during ITGA5 suppression-induced tumor cell anoikis.

## DISCUSSION

Identifying the functional role and underlying mechanism of miRNAs aberrantly expressed in tumor tissues would provide a novel insight for the exploration of miRNA-based diagnosis and therapeutics. Although miR-26a may function as an oncogene in certain cell type contexts like malignant glioma, cholangiocarcinoma and chronic lymphocytic leukemia [35-37], accumulating evidence demonstrates that in most of detected malignant tumors, miR-26a exhibits tumor-inhibitory properties. Mendell's group first reported that the administration of virus-mediated miR-26a potently eliminates tumor burden in a transgenic mouse model [24]. Our analysis of mRNA array data from the Gene Expression Omnibus (GEO) repository show that downregulation of miR-26a is a frequent event during the metastasis of HCC in patients (Figure 1). This finding is consistent with previously reported data in other cohorts, further confirming the correlation of miR-26a expression and the development and progression of liver cancer. Although numerous evidence support the role of miR-26a as a critical antitumor molecule in HCC, the underlying mechanism remains unclear. Two recent reports show that both IL-6-Stat3 and HGF-Met oncogenic signaling pathways are negatively regulated by miR-26a in HCCs, suggesting that downregulation of miR-26a promotes proliferation, migration and invasion of tumor cells *in vitro* and *in vivo* [25, 26]. This supports the notion that a single miRNA can act via the pleiotropic regulation of multiple effectors. Provided that anoikis is a major barrier of tumor cell metastasis, our study identified a novel and crucial role of miR-26a during HCC pathogenicity.

Anoikis, a detachment-induced type of apoptosis, exerts as a barrier to cancer metastasis. Resistance to anoikis compromises tumor cells to survive in the systemic circulation and facilitates their metastasis to distant organs [7]. It has been demonstrated that patients with circulating tumor cells in the peripheral blood have a much worse prognosis compared with patients without the circulating cells [38]. And it is widely accepted that metastasis is a late event in cancer progression. However, recent evidence from mouse models and clinical patients showed the existence of disseminated tumor cells in preinvasive stages of tumor progression [39, 40]. Therefore, to deeply uncover the mechanism of anoikis resistance would be potential to decrease metastasis and thereby improve patient survival. In this study, we discovered that overexpression of miR-26a sensitizes resistant tumor cells to anoikis (Figure 2). Using a combination of bioinformatic prediction and clinical sample analysis, we narrowed down ITGA5 as the putative target gene of miR-26a during anoikis (Figures 3, S1 and S2). This finding was validated *in vitro* and *in vivo* by reconstituting ITGA5 expression in HCC cells (Figures 4 and 5). We show that AKT activation is dependent on ITGA5 expression and is required to override anoikis in HCC cells (Figure 6). Interestingly, we show that ERK1/2 activation, previously reported to play a critical role during anoikis resistance of non-transformed cells like mammary epithelial cells and mesenchymal stem cells *in vitro* [41, 42], is dispensable for anoikis resistance in HCC cells (Figure 6). This is in agreement with previous studies using high throughput screening that AKT signaling pathway is critical for the resistance of tumor cells to anoikis [34]. It is important to note that in both ITGA5 high and low HCC cells derived from the same parental cell lines do not exhibit apoptosis differences under adherent conditions (data not shown) therefore indicating that ITGA5 expression specifically correlates with anoikis.

It is well documented that ITGA5 and integrin β1 (ITGB1) form heterodimers to mediate cell adhesion to fibronectin (FN). ITGA5 promotes tumor cell adhesion and migration through activating focal adhesion kinase (FAK) [43]. Blocking this heterodimer's function by an antibody (Volociximab) or a non–RGD-based peptide inhibitor (ATN-161) significantly inhibits growth and metastasis of breast cancer cells *in vitro* and *in vivo* [44]. Although ITGA5 is a promising candidate in cancer treatment, its expression pattern and function in neoplastic cells are still elusive. Here, we discovered that the expression levels of ITGA5 are much higher in HCC compared to adjacent normal liver tissues (Figure 4E) and correlates with poor prognosis of clinical patients (Figure 4G). A similar phenomenon is also detected in colorectal cancers (Figure S4). All these findings strongly suggest that ITGA5 is an oncogenic driver in solid tumors. Intriguingly, we discovered that there is no obvious correlation between ITGA5 and FN expression level in human hepatocellular carcinomas (Figure 4H). This suggests that when tumor cells detach from ECM and come into circulation system, ITGA5 may still have some uncharacterized functions to maintain survival of tumor cells which is independent of FN stimulation. In the present study, our gain-of-function and loss-of-function experiments showed that ITGA5 is a master regulator of anoikis in tumor cells (Figures 4 and 5). It has been important to determine the inhibitory effect of miR-26a on anoikis of HCC cells *in vitro* and *in vivo*.

Despite the importance of anoikis resistance during tumor metastasis, no appropriate system is currently available to accurately measure anoikis in cancer cells *in vivo*. This is because the dwell time of circulating tumor cells (CTCs) in the circulation system is only several hours [27]. Although bioluminescence imaging (BLI) is a powerful tool for localizing and quantifying metastatic tumor growth, the spatial resolution of BLI is relatively poor and the optical signal propagation through living tissue compromises sensitivity and complicates accurate measurements. Gaussia luciferase (Gluc) is a naturally secreted luciferase from the marine copepod Gaussia princeps and is over thousands folds more sensitive than firefly and renilla luciferases in mammalian cells [28]. Since Gluc is secreted, its concentration in the blood correlates with expression level *in vivo* thereby can measure survival and early growth of metastatic tumors with increased sensitivity compared to BLI [29]. Here, we genetically modified all the cell lines with this secreted luciferase gene. Base on the blood Gluc assay, we dynamically and accurately evaluated the sensitivity of anoikis in tumor cells with the different levels of ITGA5. In line with the dwell time of CTCs in clinical patients, Gluc activity in the blood of nude mice can only be measured within 10 hours (Figures 2E and 4D). Expectedly, miR-26a overexpressed HCC cells performed a significantly decrease in Gluc activity compared with the control cells (Figure 2E). This assay helps us to accurately evaluate the impact of miR-26a on anoikis sensitivity of HCC cells *in vivo*.

Our results provide the first evidence that, in aggressive HCCs, miR-26a governs tumor cell anoikis sensitivity, at least in part, by downregulating the expression level of ITGA5. Our findings combined with that of others indicate that miR-26a sits atop a regulatory pathway that inhibits multiple steps of the metastatic cascade, altering both the capacity of cancer cells to detach from a primary tumor and the ability of already disseminated neoplastic cells to survive in circulation system. The pleiotropic anti-metastatic capabilities of miR-26a seem to position this tumor suppressive miRNA as a critical safeguard against the acquisition of metastatic competence. Consequently, it is plausible that therapeutic strategies directed toward restoring miR-26a function would prove to be clinically useful in limiting metastatic progression in HCC.

## MATERIALS AND METHODS

### Antibodies and reagents

The primary antibodies used were: anti-ITGA5 antibody (sc-136224) from Santa Cruz Biotechnology (Santa Cruz, CA, USA); anti-PARP antibody (#9532), anti-phospho(Ser473) and anti-total Akt antibody (#4060 and #9272, respectively), anti-phospho(Thr202/Tyr204) and anti-total Erk1/2 antibody (#9101 and #9102, respectively) from Cell Signaling Technology (Danvers, MA, USA); anti-beta-actin antibody (A5441) and Poly(2-hydroxyethyl methacrylate) (polyHEMA, P3932) from Sigma–Aldrich (St. Louis, MO, USA). The secondary antibodies were IR-Dye 800-conjugated secondary antibodies (Rockland, Gilbertsville, PA, USA). The Akt inhibitor (124005) and the ERK inhibitor (328007) were both from Calbiochem (La Jolla, CA, USA).

### Cell culture

Human embryonic kidney cell lines (HEK-293 and HEK-293T) and human liver cancer cell line (BEL-7404, HepG2 and SMMC-7721) were obtained from the Type Culture Collection of the Chinese Academy of Sciences. Human liver cancer cell line FHCC-98 was preserved in our lab. The immortalized hepatocyte cell line L-02 was also obtained from the Type Culture Collection of the Chinese Academy of Sciences. Adherent cultures of HEK-293, HEK-293T and HepG2 cell lines were maintained in Dulbecco's modified Eagle's medium (DMEM; Gibco BRL, USA) supplemented with 10% fetal calf serum (FCS; Gibco BRL, USA), 2 mM L-glutamine and antibiotics (complete medium). The FHCC-98, SMMC-7721, L-02 and BEL-7404 cell lines were maintained in RPMI-1640 medium (Gibco BRL, USA) supplemented with 10% FCS and antibiotics. All the cells were incubated in a humidified atmosphere of 5% CO_2_ in air at 37°C.

Poly-HEMA (Sigma–Aldrich; St. Louis, MO, USA), a non-adhesive substratum, was prepared using a solution of 10 mg/ml in 95% ethanol and was added to culture plates at 1.5×10^−1^ ml/cm^2^ and allowed to evaporate to dryness at room temperature. The plates were then washed twice with HBSS and HCC cells were plated at a concentration of 1.0×10^5^ cells/ml and in differentiating media. 48 hours later, the cells were harvested for analysis.

### Western blot

Cells were lysed for 20 minutes in ice-cold radio-immuno-precipitation assay buffer (RIPA; 20 mM Tris-HCl pH 7.4, 137 mM NaCl, 10% glycerol, 1% Triton X-100, 0.5% sodium deoxycholate, 0.1% SDS, 2 mM EDTA pH 8, 2 mM vanadate, 1 mM PMSF and a cocktail of protease inhibitors). Cell lysate was cleared by centrifugation and an appropriate sample buffer was added. Samples were subjected to SDS-PAGE, immunoblotted with the appropriate primary antibodies (anti-ITGA5, 1:200; anti-phospho-Akt 1:1000; anti-total-Akt, 1:1000; anti-phospho-Erk1/2 1:1000; anti-total-Erk1/2, 1:1000; anti-PARP, 1:1000; or anti-beta-actin, 1:4000), incubated with the corresponding IR-Dye 800-conjugated secondary antibodies and detected using a LI-COR Odyssey Infrared Imaging System (LI-COR, Lincoln, NE, USA). The intensity of the bands was analyzed using the Image J software.

### RNA isolation, reverse transcription and real-time polymerase chain reaction

Total RNA was extracted using TRIzol (Invitrogen, Carlsbad, CA, USA) according to the manufacturer's instructions. Reverse transcription (RT) for gene expression or miRNA expression was carried out by using the PrimeScript™ RT Master Mix (TaKaRa, Japan; 1μg total RNA) or the miScript Reverse Transcription Kit (Qiagen, Germany; 1μg total RNA), respectively.

For measuring gene and miRNA expression, the quantitative real-time PCR (qPCR) was conducted using the SYBR Green dye (TaKaRa, Japan) according to the manufacturer's protocol. The following primers were used for the analysis: ITGA5, forward primer, 5′-GTCGGGGGCTTCAACTTAGAC-3′, reverse primer, 5′-CCTGGCTGGCTGGTATTAGC-3′; GAPDH (as an endogenous control), forward primer, 5′-TCACCAGGGCTGCTTTTAAC-3′, reverse primer, 5′-GACAAGCTTCCCGTTCTCAG-3′; miR-26a, forward primer, 5′-TTCAAGTAATCCAGGATAGGCT-3′, reverse primer, Universal Primer (QIAGEN, Germany); U6-snRNA, forward primer, RNU6B_2 miScript Primer (QIAGEN, Germany), reverse primer, Universal Primer (QIAGEN, Germany). Real-time PCR was performed in triplicate on CFX96 Real Time PCR Detection System (Bio-Rad, USA), the 2^-ΔΔCT method was used to determine the relative gene expression, and mature miRNA were normalized to U6-snRNA.

### Constructs

#### luc-UTR vectors

The ITGA5 3′UTR was cloned into the EcoRI and PstI sites of a modified pGL3 luciferase vector as previously described [18] with forward primer, 5′- GTCCTCCCAATTTCAGACTCCCAT-3′, and reverse primer, 5′-GCTTCAGGGAGGCTGGGGCC-3′. The PCR products with the appropriate primers generated inserts with point substitutions in the miRNA complementary sites to generate the Mut-ITGA5-3′UTR vector as a mutant control. The constructs were sequence verified.

#### Constructs for miR-26a overexpression

Synthesized RNA duplexes of scramble miRNA (Negetive Control, NC) and miR-26a were obtained from GenePharma (Shanghai, China). To construct a lentiviral vector expressing miR-26a, pre-miR-26a-2 and 150bp of flanking sequence was amplified with forward primer, 5′- ATCGTAAGGGTGGACAA-3′ and reverse primer, 5′-AGCTCTCAACCCCTGCA-3′. A PCR fragment was inserted into the pENTR^TM^3C vector (Invitrogen, Carlsbad, CA, USA) using EcoR^I^and Xho^I^sites. The pLenti6.3-miR-26a vector was constructed using Gateway LR Clonase ^II^ Enzyme Mix (Invitrogen, Carlsbad, CA, USA) according to the manufacturer's instructions. The constructs were sequence verified.

#### Constructs for ITGA5 overexpression and Knockdown

The ITGA5 coding sequence was amplified from cDNA using primers: 5′- ATGGGGAGCCGGACGCCAGA-3′ and 5′-TCAGGCATCAGAGGTGGCTG-3′. The fragment was cloned into pENTR^TM^3C vector using Kpn^I^and EcoR^V^ digestion, and inserted into pLenti6.3 vector as pLenti6.3-miR-26a described before.

Vector used for RNAi was constructed by inserting a synthesized 58-mer oligonucleotide containing a specific sequence for ITGA5. The target sequence for ITGA5 was: 5′-AACTCTGGTCACATATAGGAG-3′. The oligos were resuspended in annealing buffer and heated to 95°C for 5 minutes and then cooled to room temperature to generate double-stranded DNA. The double-stranded oligos were clone into lentiviral vector pLKO.1. The vector of pLKO.1 GFP shRNA was obtained from Addgene (Cambridge, MA, USA; Plasmid 30323). The constructs were sequence verified.

#### Viral packaging and cell infection

Using the VSVG-based package system 8μg of vectors were added to tubes containing the Lipofectamine 2000 transfection reagent (Invitrogen, Carlsbad, CA, USA). The plasmids were transfected into HEK-293T cells; after 48 and 72 hours, the viral supernatants were filtered through a 0.45μm cellulose acetate filter and concentrated by centrifugation at 50,000 g for 90 minutes. The viral supernatants were then used to infect the human HCC cells in the presence of polybrene (Sigma, St. Louis, MO, USA). Lastly, the infected HCC cells were selected with 1-10 mg/l Blasticidin S (Sigma, St. Louis, MO, USA) or 1 mg/l puromycin (Sigma, St. Louis, MO, USA).

#### *In vitro* luciferase assay

HEK-293 cells of 50% confluence in 48 well plates were transfected using Lipofectamine 2000 (Invitrogen, Carlsbad, CA, USA). The mimics of miR-26a or miR-NC (20 pmol) along with firefly luciferase reporter gene construct (100ng) and a Renilla luciferase construct (5 ng; for normalization) were co-transfected per well. Firefly and Renilla luciferase activities were assessed using the Dual-Glo luciferase assay system (Promega, Madison, WI, USA) in accordance with the manufacturer's instructions. Luminescence readings were acquired using a TD 20/20 luminometer (Turner Design Inc., Sunnyvale, CA, USA). Sample values were compared to the reference value of cells transfected miR-NC. The experiments were performed in triplicate.

#### Assessment of anoikis *in vitro*

HCC cells (1×10^6^) were seeded in poly-HEMA pre-coated 6-well plates at 37°C for 48 hours. The cells were harvested and apoptosis assessed by two methods. Exposure of phosphatidylserine on the outer cellular membrane was evaluated by flow cytometry using an Annexin V-FITC/propidium iodide (PI) kit (Biovision, Palo Alto, CA, USA). Caspase-3 activity was assayed using a caspase-3 activity kit (Beyotime Institute of Biotechnology, Jiangsu, China) and calculated as the fold increase of caspase-3 activity over that of the Luc (negative) control.

#### Assessment of anoikis *in vivo*

Six-week-old BALB/c nude mice were purchased from the Model Animal Research Center of Nanjing University. All animal procedures were performed in accordance to the criteria outlined in the “Guide for the Care and Use of Laboratory Animals” prepared by the National Academy of Sciences and published by NIH. BEL-7404 cells were infected with Lv-Gluc (Gauss Luciferase) to produce cell strain named BEL-7404 Gluc, which stably secreted Gluc outside the cells. BEL-7404-Gluc cells were infected with appropriate lentiviruses to produce BEL-7404 Gluc_Luc, BEL-7404 Gluc_miR-26a, BEL-7404 Gluc_shGFP and BEL-7404 Gluc_shITGA5. Treated cells were injected into BALB/c nude mice through the tail vein (1 × 10^6^ cells/mouse, five mice/group). Half an hour after injection, blood samples were withdrawn by making a small incision in the tail of awake mice. Typically, 5 μl blood was added to 1 μl 20 mM EDTA and Gluc activity was measured using a TD 20/20 luminometer (Turner Design Inc., Sunnyvale, CA, USA) which was set to inject 100 μl 100 μM coelenterazine (GOLDBIO, St. Louis, MO, USA) in DMEM and to acquire photon counts for 10 sec. Blood samples were withdrawn and photon was monitored every 2 hours for a total period of 10 hours.

#### Bioinformatic analysis

TargetScan6.2 (http://www.targetscan.org) and PicTar (http://pictar.mdc-berlin.de) were used to predict miR-26a targets. Gene ontology analysis and pathway enrichment analysis were done in DAVID website (http://david.abcc.ncifcrf.gov). Potential miR-26a targeted genes associated with focal adhesion were analyzed and visualized by GenMAPP software (Version 2.1). Pre-miR-26a-1, pre-miR-26a-2 and mature miR-26a expression profiling in HCC was downloaded from a normalized GEO dataset (GSE6857). In Figure 3B, 4E and S2, the expression profiling of mRNAs was downloaded from another correlative normalized GEO dataset (GSE14520). The above mentioned dataset (GSE14520) was also used to compare the ITGA5 expression between non-tumor tissues and tumor tissues, and P-value was calculated using SPSS 13.0 software. In Figure 4F-H, the expression level of mRNA and the clinical data were all from The Cancer Genome Atlas (TCGA) liver hepatocellular carcinoma normalized dataset. In Figure S4, the expression level of ITGA5 and the clinical data in colorectal cancers were from a normalized GEO dataset (GSE 17538).

#### Statistical analysis

Statistical analysis was performed using SPSS 13.0 for Windows. All of the experiments were conducted at least in triplicate, and the results were expressed as the means ± s.d.. Two-tailed Student's t-test were used to evaluate statistical significance. The significance of associations between gene expression values was judged via a test statistic based on Pearson product-moment correlation coefficient. Log-rank (Mantel-Cox) test was used for the Kaplan-Meier survival analysis. Differences were considered significant when *P* < 0.05 (*), *P* < 0.01 (**) or *P* < 0.001 (***).

## SUPPLEMENTARY MATERIAL FIGURES


