# Supplementary material for: MicroRNA-26a promotes anoikis in human hepatocellular carcinoma cells by targeting alpha5 integrin
Source: Oncotarget. 2014 Dec 10;6(4):2277–89. doi: 10.18632/oncotarget.2956 (PMC4385851; doi:10.18632/oncotarget.2956)
Supplement: Supplementary file 1 [file oncotarget-06-2277-s001.pdf]

MicroRNA-26a promotes anoikis in human hepatocellular carcinoma cells by targeting alpha5 integrin

Supplementary Material

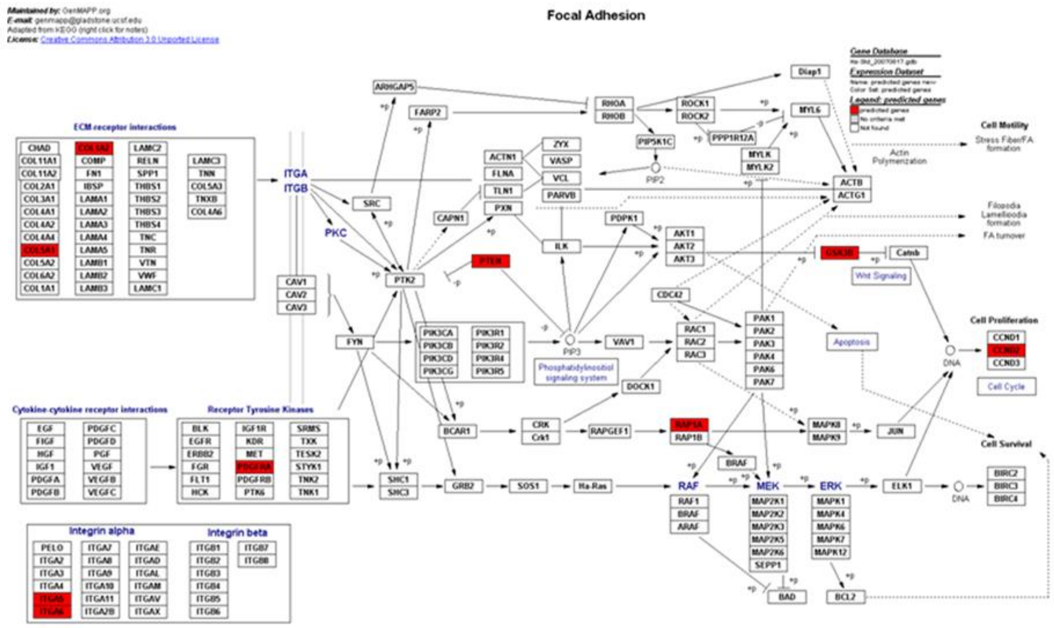

Supplementary figure S1: Potential miR-26a targeted genes associated with focal adhesion were highlighted by GenMAPP.

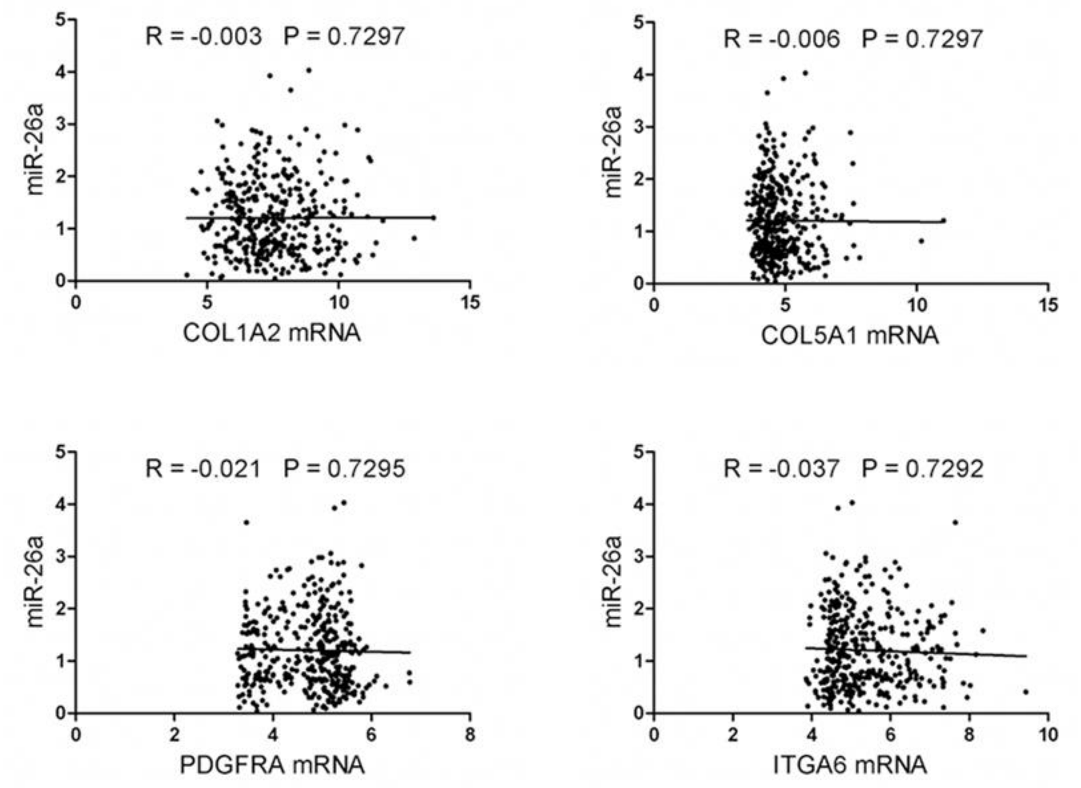

Supplementary figure S2: No correlation of the other (except ITGA5) mRNA and miR-26a in human HCC tissues.

The Pearson product-moment correlation coefficient and significance levels are indicated. Expression levels of mRNA and miR-26a were from two correlative normalized GEO datasets (GSE14520 & GSE6857).

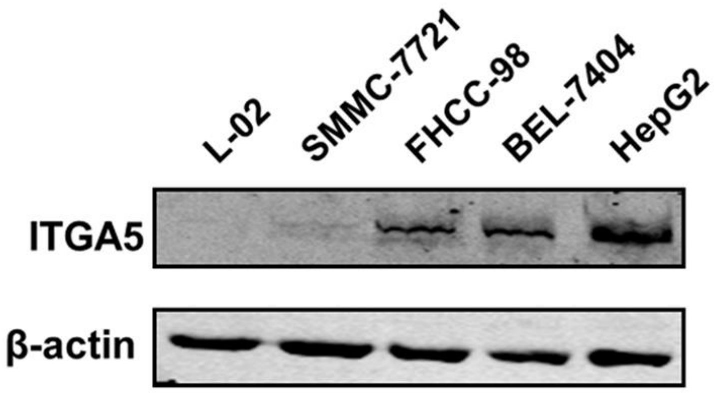

Supplementary figure S3: Western blot analysis showing ITGA5 expression at the protein level in different human HCC cell lines and normal hepatocyte-derived cell line (L-02).

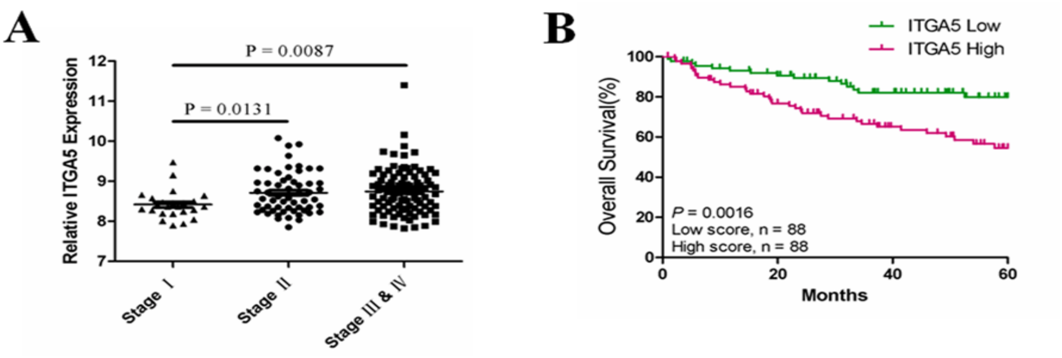

Supplementary figure S4: ITGA5 could also be an oncogenic driver in development and progression of colorectal cancers.

(A) Relative ITGA5 levels assessed by mRNA array in colorectal cancer (CRC) tissues stratified according to the stages of CRC. (B) Kaplan-Meier graph representing the probabilities of overall survival in colorectal cancer patients stratified according to the expression levels of ITGA5. Expression levels of ITGA5 mRNA were from a normalized GEO dataset (GSE17538).
